# Supplementary material for: Testing and Prescribing Vitamin B12 in Swiss General Practice: A Survey among Physicians
Source: Nutrients. 2021 Jul 29;13(8):2610. doi: 10.3390/nu13082610 (PMC8398177; doi:10.3390/nu13082610)
Supplement: Supplementary file 1 [file nutrients-13-02610-s001.zip › Nu_Survey_B12_Supplementary_Data.pdf]

Supplement Figure S1: Scree plot for the B12 testing attitude score. Principal component variances are plotted against their rank. The plot is highly suggestive for unidimensionality.

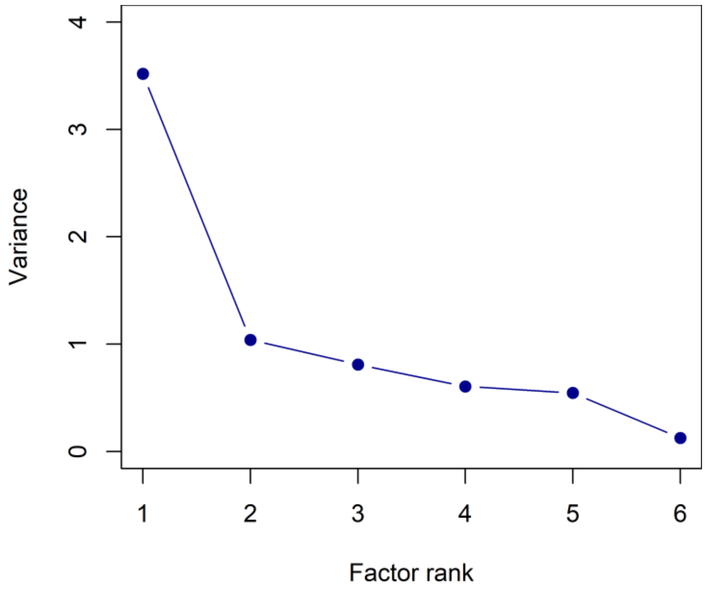

Supplement Table S1: Results of multivariable linear regression for the B12 testing attitude score.

Variable	Estimate (95% CI [1])	p-value
Intercept	13.90 (11.02, 16.78)	< 0.001
Gender: male (reference: female)	1.02 (0.15, 1.90)	0.02
Experience as general practitioner in years	0.04 (-0.01, 0.09)	0.10
< 50% of prescriptions as placebo	1.78 (0.31, 3.25)	0.02
< 50% of prescriptions on patients' request	-0.79 (-2.61, 1.03)	0.39
< 50% of testing on patients' request	1.48 (0.40, 2.56)	0.01
Practices CAM [2]	-0.86 (-2.88, 1.16)	0.40
Practices psychosomatic medicine	0.71 (-1.24, 2.65)	0.48

[1] confidence interval.
[2] complementary and alternative medicine
