# Supplementary material for: Testing and Prescribing Vitamin B12 in Swiss General Practice: A Survey among Physicians
Source: Nutrients. 2021 Jul 29;13(8):2610. doi: 10.3390/nu13082610 (PMC8398177; doi:10.3390/nu13082610)
Supplement: Supplementary file 1 [file nutrients-13-02610-s001.zip › Nu_Survey_B12_Supplementary_File_S1.pdf]

Universität Zürich UZH

Institut für Hausarztmedizin

Umfrage zum Thema Vitamin B12 in der Hausarztmedizin

Herzlich willkommen

Liebe Kolleginnen und Kollegen

Vitamin B12 ist ein populäres aber noch nicht in jeder Hinsicht gut erforschtes Thema. Das Institut für Hausarztmedizin der Universität Zürich führt diese Umfrage durch zur Eingrenzung von relevanten Fragestellungen für zukünftige Studien. Hierbei zählen wir auf Ihre wertvolle Unterstützung.

Wir haben die Umfrage sehr kurz gehalten (ca. 8 Minuten) und als Dankeschön haben Sie zudem die Möglichkeit, an der Verlosung von 3 x 500.- teilzunehmen.

Herzlichen Dank für Ihre Teilnahme und freundliche Grüsse

Katarina Bardheci
Stefan Markun
Jakob Martin Burgstaller
Levy Jäger

Disclaimer

Diese Umfrage wurde mit SurveyMonkey® erstellt. Der Anbieter erfüllt alle Anforderungen des Datenschutzes gemäss aktuell gültiger Datenschutz-Grundverordnung DSGVO (hier finden Sie die Datenschutzrichtlinie von Surveymonkey®). Die Studie wurde durch das Institut für Hausarztmedizin der Universität Zürich initiiert. Die Daten werden lokal durch das Studienteam wissenschaftlich ausgewertet und eine Weitergabe/Vermarktung erfolgt zu keinem Zeitpunkt. Es besteht keine Einflussnahme oder Finanzierung durch die Pharmaindustrie. Bei allen beteiligten Personen bestehen keine Interessenskonflikte. Die Resultate der Studie sollen öffentlich publiziert werden (hier besteht volle Anonymität).

Wenn Sie an dieser Umfrage teilnehmen möchten, geben Sie hier bitte den im Einladungs-Email angegebenen **fünfstelligen Code** ein:

1. Wie stark stimmen Sie zu, dass bei der Abklärung der untenstehenden klinischen Situationen die Bestimmung des Vitamin-B12-Status **immer** indiziert ist?

(Der Einfachheit halber haben wir jeweils die männliche Form angewandt stellvertretend für Patient und Patientin)

	Ich stimme überhaupt nicht zu	Ich stimme eher nicht zu	Ich stimme teilweise zu	Ich stimme eher zu	Ich stimme vollständig zu
Verdacht auf periphere Polyneuropathie	○	○	○	○	○
Anämie	○	○	○	○	○
Idiopathische Müdigkeit	○	○	○	○	○
Kognitive Beschwerden	○	○	○	○	○
Depressive Symptome	○	○	○	○	○
Check-up asymptomatischer Patienten	○	○	○	○	○

2. Im Falle, dass die von Ihnen verwendeten Laborparameter einen Vitamin B12-Status im **Graubereich** (weder eindeutiger Mangel noch eindeutige Suffizienz) zeigen, wann würden Sie Vitamin B12 **üblicherweise** (empirisch) verabreichen?

- ☐ Bei Verdacht auf periphere Polyneuropathie
- ☐ Bei Anämie
- ☐ Bei idiopathischer Müdigkeit
- ☐ Bei kognitiven Beschwerden
- ☐ Bei depressiven Symptomen
- ☐ Beim Check-up asymptomatischer Patienten
- ☐ Ich verwende/behandle keinen Graubereich
- ☐ Bei folgenden (weiteren) Situationen mit Vitamin-B12-Status im Graubereich

3. Von allen Fällen, in denen Sie den **Vitamin-B12-Status** im letzten Jahr in Ihrer Sprechstunde **bestimmt haben**, wählen Sie die **drei häufigsten** klinischen Situationen, gemäss Ihrer Einschätzung:

- [ ] Verdacht auf periphere Polyneuropathie
- [ ] Anämie
- [ ] Idiopathische Müdigkeit
- [ ] Kognitive Beschwerden
- [ ] Depressive Symptome
- [ ] Screening bei asymptomatischen Patienten
- [ ] Bekanntes Risiko für Malnutrition/Malabsorption (z.B. Veganer oder bei funktionellem Kurzdarm)
- [ ] Haarausfall/Aphthen
- [ ] Andere Situationen

4. Von allen Fällen, in denen Sie **Vitamin B12** im letzten Jahr in Ihrer Sprechstunde **verschrieben haben**, wählen Sie die **drei häufigsten** klinischen Situationen, gemäss Ihrer Einschätzung:

- [ ] Verdacht auf periphere Polyneuropathie
- [ ] Anämie
- [ ] Idiopathische Müdigkeit
- [ ] Kognitive Beschwerden
- [ ] Depressive Symptome
- [ ] Bei asymptomatischen Patienten (nach Screening/präventiv)
- [ ] Bekanntes Risiko für Malnutrition/Malabsorption (z.B. Veganer oder bei funktionellem Kurzdarm)
- [ ] Haarausfall/Aphthen
- [ ] Andere Situationen

5. Wie gehen Sie bei der Bestimmung des Vitamin-B12-Status üblicherweise vor (Stufendiagnostik)? Bitte teilen Sie untenstehende Laborparameter nach Verwendung als Firstline-Test (bei der ersten Blutentnahme) oder Secondline-Test (bei der zweiten Blutentnahme oder Nachbestellung) ein.

	Firstline	Secondline	Bestimme ich nicht oder nur ausnahmsweise
Serum-Vitamin B12	○	○	○
Holotranscobalamin	○	○	○
Homocystein	○	○	○
Methylmalonsäure	○	○	○

6. Wie häufig erfolgen **Laboruntersuchungen** des Vitamin-B12-Status in Ihrer Sprechstunde **ausschliesslich** aufgrund von ausdrücklichem Wunsch des Patienten, gemäss Ihrer Einschätzung?

0% (nie, Vitamin-B12-Laboruntersuchungen erfolgen ausschliesslich auf meine Empfehlung) — 50% — 100% (immer, Vitamin-B12-Laboruntersuchungen erfolgen ausschliesslich auf Wunsch des Patienten)

7. Wie häufig erfolgen **Behandlungen** mit Vitamin B12 in Ihrer Sprechstunde **ausschliesslich** aufgrund von ausdrücklichem Wunsch des Patienten, gemäss Ihrer Einschätzung?

0% (nie, B12 Behandlungen erfolgen ausschliesslich auf meine Empfehlung) — 50% — 100% (immer, B12 Behandlungen erfolgen ausschliesslich auf Wunsch des Patienten)

8. In welchem Verhältnis verschreiben Sie Vitamin B12 parenteral (z.B. als intramuskuläre Injektionen) im Gegensatz zu anderen Darreichungsformen, gemäss Ihrer Einschätzung?

0% parenteral (d.h. immer oral) — 50% — 100% parenteral (z.B. immer als Injektion)

9. Wie häufig verschreiben Sie Vitamin B12 mit geringer Wirksamkeitserwartung (vorwiegend als Placebo), gemäss Ihrer Einschätzung?

0% (nie als Placebo) — 50% — 100% (immer als Placebo)

Umfrage zum Thema Vitamin B12 in der Hausarztmedizin

Teilnehmerangaben

10. Geschlecht

- ◯ Weiblich
- ◯ Männlich

11. Anzahl Jahre Berufserfahrung als Ärztin/Arzt

0 — 40 oder mehr ☐

12. Wo sind Sie hauptsächlich tätig?

- ◯ Einzelpraxis
- ◯ Gruppenpraxis (mit mindestens 1 weiteren Ärztin/
- ◯ Arzt) Anderes Arbeitsumfeld

13. Welche(n) der folgenden Facharzttitel, Fähigkeitsausweis(e)/Interdisziplinären Schwerpunkt(e) haben Sie?

- ☐ Facharzt Allgemeine Innere Medizin/Innere Medizin/Allgemeinmedizin
- ☐ Facharzt Praktische Ärztin/Praktischer Arzt
- ☐ Kein Facharzttitel
- ☐ Fähigkeitsausweis(e) in Methoden der Alternativmedizin (Akupunktur - Chinesische Arzneitherapie - TCM, antroposophisch erweiterte Medizin, Homöopathie, Phytotherapie)
- ☐ Fähigkeitsausweis(e) in medizinscher Hypnose und/oder delegierter Psychotherapie und/oder interventioneller Psychiatrie und/oder Schwerpunkt in psychosomatischer und psychosozialer Medizin
- ☐ Fähigkeitsausweis in Schlafmedizin

14. Wie arbeiten Sie hauptsächlich?

- ◯ Als Hausärztin/Hausarzt
- ◯ Als Spezialistin/Spezialist (anderer Facharzttitel)
- ◯ Zu gleichen Teilen als Hausärztin/Hausarzt und Spezialistin/Spezialist (anderer Facharzttitel)
- ◯ Anderes

15. Die Evidenz zur Wirksamkeit von Vitamin B12 bei idiopathischer Müdigkeit ist mehr als dürftig. Wären Sie daran interessiert, mit Ihrer Praxis an einer randomisierten Studie zur Wirksamkeit von Vitamin B12 bei idiopathischer Müdigkeit teilzunehmen?

- ◯ Ja, das würde mich (eventuell) interessieren
- ◯ Nein

Hier können Sie zusätzlich eigene Ideen für wissenschaftliche Fragestellungen oder sonstiges Feedback zu dieser Umfrage angeben.

16. Möchten Sie an der Verlosung von 3 x 500.- teilnehmen?

- ◯ Ja, gerne
- ◯ Nein, danke

17. Möchten Sie die Ergebnisse der Umfrage per E-Mail erhalten?

- ◯ Ja, gerne
- ◯ Nein, danke

Universität Zürich[UZH]
Institut für Hausarztmedizin

Umfrage zum Thema Vitamin B12 in der Hausarztmedizin

Sie haben angegeben, dass Sie an der Verlosung oder an einer Folgestudie oder an den Umfrageergebnissen interessiert sind. Dafür benötigen wir Ihre Kontaktdaten. Um die Anonymität Ihrer Beantwortungen zu gewährleisten, werden Ihre Kontaktdaten von Ihren Antwortdaten getrennt.

18. Name, Vorname

19. Adresse

20. Postleitzahl

21. Ort

22. E-Mail-Adresse

Universität Zürich[UZH]

Institut für Hausarztmedizin

Umfrage zum Thema Vitamin B12 in der Hausarztmedizin

Die Umfrage ist beendet. Wir bedanken uns herzlich für Ihre Teilnahme!
