# Supplementary material for: Testing and Prescribing Vitamin B12 in Swiss General Practice: A Survey among Physicians
Source: Nutrients. 2021 Jul 29;13(8):2610. doi: 10.3390/nu13082610 (PMC8398177; doi:10.3390/nu13082610)
Supplement: Supplementary file 1 [file nutrients-13-02610-s001.zip › Nu_Survey_B12_Supplementary_File_S2.pdf]

Universität Zürich[UZH]
Institut für Hausarztmedizin

Survey on vitamin B12 in general practice

Welcome

Dear colleagues,

Vitamin B12 is a popular topic, but not well studied in every aspect yet. The Institute of Primary Care of the University of Zurich is conducting this survey to evaluate relevant aspects for future studies. We hope we can count on your support.

The survey can be answered very quickly (in around 8 min) and to thank you for your support, you will be eligible to take part in a price draw of 3 x 500 CHF.

Many thanks for your participation and best regards,

Katarina Bardheci
Stefan Markun
Jakob Martin Burgstaller
Levy Jäger

Disclaimer

This survey is conducted using SurveyMonkey®. SurveyMonkey® is compliant with data protection law according to DSGVO (see here to find the data protection policy of Surveymonkey®). The study was initiated by the Institute of Primary Care of the University of Zurich. Data are stored and analyzed by the study team and not shared with third parties. This study is independent from the pharmaceutical industry and the study team has no conflicts of interest. Results from this study will be published but responses from individuals will remain fully anonymized.

If you want to participate in this survey, please enter your 5-digit code from the invitation email here:

____

1. How strongly do you agree that vitamin B12 testing is **always** needed for evaluation of the clinical situations stated below?

	I fully disagree	I rather disagree	I partially agree	I rather agree	I fully agree
Suspected peripheral polyneuropathy	○	○	○	○	○
Anemia	○	○	○	○	○
Idiopathic fatigue	○	○	○	○	○
Cognitive complaints	○	○	○	○	○
Depressive symptoms	○	○	○	○	○
Check-up of asymptomatic patients	○	○	○	○	○

2. In the event of having a vitamin B12 status in the **“borderline” range** according to the used laboratory measures (neither a clear deficiency nor a clear sufficiency), when would you **usually** administer vitamin B12 (empirically)?

- ☐ For suspected peripheral polyneuropathy
- ☐ For anemia
- ☐ For idiopathic fatigue
- ☐ For cognitive complaints
- ☐ For depressive symptoms
- ☐ For check-up of asymptomatic patients
- ☐ I do not use/treat "borderline" ranges
- ☐ In the following (other) situations with a vitamin B12 status in the "borderline" range:

3. Please choose the **three most common** clinical situations of all cases you **tested** the **vitamin B12 status** in your consultation hours in the last year (according to your best estimation):

- [ ] Suspected peripheral polyneuropathy
- [ ] Anemia
- [ ] Idiopathic fatigue
- [ ] Cognitive complaints
- [ ] Depressive symptoms
- [ ] Screening of asymptomatic patients
- [ ] Known risk for malnutrition/malabsorption (e.g., vegan or with short bowel syndrome)
- [ ] Hair loss/oral aphthae
- [ ] Other situations

4. Please choose the **three most common** clinical situations of all cases you **prescribed vitamin B12** to in your consultation hours in the last year (according to your best estimation):

- [ ] Suspected peripheral polyneuropathy
- [ ] Anemia
- [ ] Idiopathic fatigue
- [ ] Cognitive complaints
- [ ] Depressive symptoms
- [ ] Asymptomatic patients (after screening/preventive)
- [ ] Known risk for malnutrition/malabsorption (e.g., vegan or with short bowel syndrome)
- [ ] Hair loss/oral aphthae
- [ ] Other situations

5. What is your standard procedure for testing vitamin B12 status (step-by-step diagnosis)? Please categorize the laboratory parameters below depending on use as first-line test (first blood sample) or as second-line test (second blood sample or reorder).

	First-line	Second-line	I don't use or only exceptionally use this test
Serum vitamin B12	○	○	○
Holotranscobalamin	○	○	○
Homocysteine	○	○	○
Methylmalonic acid	○	○	○

6. How often do you **test** the vitamin B12 status **solely** based on express request of the patients, according to your best estimation?

0% (never, vitamin B12 laboratory tests are solely based on my recommendation)	50%	100% (always, vitamin B12 laboratory tests are solely based on express request of the patients)

7. How often do you prescribe vitamin B12 **solely** based on express request of the patients, according to your best estimation?

0% (never, vitamin B12 prescriptions are solely based on my recommendation)	50%	100% (always, vitamin B12 prescriptions are solely based on express request of the patients)

8. What is the proportion of parenteral vitamin B12 prescriptions in your practice (e.g., as intramuscular injection) compared to other routes of applications, according to your best estimation?

0% parenteral (meaning always oral)	50%	100% parenteral (e.g., always as injection)

9. How often do you prescribe vitamin B12 with low confidence in effectiveness (mainly as placebo), according to your best estimation?

0% (never as placebo)	50%	100% (always as placebo)

Universität Zürich[UZH]
Institut für Hausarztmedizin

Survey on vitamin B12 in general practice

Participants' details

10. Sex

- ◯ Female
- ◯ Male

11. Years of professional experience as a medical doctor

0 ———————————— 40 or more [ ]

12. Where do you mainly work?

- ◯ Single practice
- ◯ Group practice (with at least another medical doctor)
- ◯ Different working environment

13. Which of the following medical specialist title(s), proficiency certificate(s)/interdisciplinary sub-speciality(ies) do you have?

- ☐ General internal medicine
- ☐ General medical practitioner
- ☐ Practical Medicine
- ☐ Proficiency certificates in methods of alternative medicine (Acupuncture – traditional Chinese medicine, anthroposophical medicine, homeopathy, phytotherapy)
- ☐ Proficiency certificates in medical hypnosis and/or delegated psychotherapy and/or interventional psychiatry and/or interdisciplinary sub-speciality in psychosomatic and psychosocial medicine
- ☐ Proficiency certificate in sleep medicine

14. What is your main professional activity?

- ◯ General practitioner
- ◯ Specialist (other board certification)
- ◯ In equal parts as general practitioner and specialist (other board certification)
- ◯ Other

15. There is scarce evidence of the effectivness of vitamin B12 in idiopathic fatigue. Are you interested in participating in a randomized controlled trial for the effectiveness of vitamin B12 in idiopathic fatigue?

◯ Yes, I may be interested

◯ No, thanks

Please enter here additional own ideas for scientific questions or other feedback about this survey.

16. Would you like to participate in the price draw of 3 x 500.- ?

◯ Yes, please

◯ No, thanks

17. Would you like to receive the results of this survey by email?

◯ Yes, please

◯ No, thanks

Universität Zürich[UZH]

Institut für Hausarztmedizin

Survey on vitamin B12 in general practice

You indicated your interest in the price draw, in a follow-up study, or in the results of the survey. For that reason, we need your contact details. In order to ensure anonymity of your responses, we manage your contact details separately.

18. Last name, first name

19. Address

20. ZIP code

21. City

22. Email address

Universität Zürich[UZH]
Institut für Hausarztmedizin

Survey on vitamin B12 in general practice

The survey is completed. We thank you for your participation!
